# Case Report: The third-generation sequencing confirmed a novel 7.2 Kb deletion at β-globin gene in a patient with rare β-thalassemia

**DOI:** 10.3389/fgene.2022.984996

**Published:** 2022-09-12

**Authors:** Guoxing Zhong, Zeyan Zhong, Zhiyang Guan, Dina Chen, Zhiyong Wu, Kunxiang Yang, Dan Chen, Yinyin Liu, Ruofan Xu, Jianhong Chen

**Affiliations:** ^1^ Department of Medical Genetics and Prenatal Diagnosis, Huizhou First Maternal and Child Health Care Hospital, Huizhou, Guangdong, China; ^2^ Berry Genomics Corporation, Beijing, China

**Keywords:** thalassemia, deletion, β-globin gene, breakpoint, third-generation sequencing

## Abstract

**Background:** Thalassemia was the most common monogenic diseases worldwide, which was caused by mutations, deletions or duplications in human globin genes which disturbed the synthesis balance between α- and β-globin chains of hemoglobin. There were many classics methods to diagnose thalassemia, but all of them had limitations. Although variations in the human β-globin gene cluster were mainly point mutations, novel large deletions had been described in recent years along with the development of DNA sequencing technology.

**Case report:** We present a case of 32-year-old male with abnormal hematological results. However, 23 genotypes of the most common thalassemia were not detected by two independent conventional platforms. Finally, using multiplex ligation-dependent probe amplification (MLPA), third-generation sequencing (TGS) and Gap PCR detection methods, we first confirmed the case with a novel 7.2 Kb deletion (Chr11:5222800-5230034, hg38) located at HBB gene.

**Conclusion:** Our results showed that TGS technology was a powerful tool for thalassemia breakpoint detection, had promising potentiality in genetic screening of novel thalassemia, especially for the novel deletions in globin genes.

## Introduction

Thalassemia was among the most common monogenic diseases worldwide (Taher et al., 2018). The estimated carrier rate for β-thalassemia was about 5.2% in the whole world. β-thalassemia was characterized by insufficient or missing synthesis of the β-globin chain of hemoglobin, up to now about 280 β-thalassemia mutations had been recognised, occurring in a wide range of ethnic groups, including at least 230 point mutations and at last 18 large deletions (Bain, 2020). Due to the limitations of routine molecular techniques for detecting β-globin gene variants, less than 30 deletions in HBB gene were reported as genotypes of β-thalassemia to date (Chen et al., 2022). Third-generation sequencing (TGS) was a new type of DNA high-throughput sequencing, based on long-read single-molecular real-time sequencing technology (SMRT) (Xu et al., 2020). Due to its long-read length and high detection accuracy, it was hypothesized that this technology could be applied to thalassemia carrier screening (Xiao and Zhou, 2020). In this study, we first discovered a novel 7.2 Kb deletion in the β-globin gene, which was identified precisely by the TGS technology.

## Case presentation

We present a 32-year-old male with abnormal hematological results, who was admitted to the outpatient for genetic screening of thalassemia during pregnancy examination. Hb electrophoresis and the routine hematological indices were determined during our screening program. Hematological examinations were measured by automatic hematological analyzer (BC-5180, China), which showed that he had a microcytic hypochromic anemia (Hb level of 119.0 g/L), mean cell hemoglobin (MCH) of 16.5 pg and a mean cell volume (MCV) of 63.8 fl (Table 1). The Hb electrophoresis was performed and quantified by automatic capillary electrophoresis (Sebia, France), which showed that the high HbA2 (7.0%), high HbF (3.0%), and the HbA was 90.0% (Table 1), iron deficiency was excluded. His wife had normal Hb and normal MCV and MCH (Table 1). The study was approved by the Medical Ethics Committee of Huizhou First Maternal and Child Health Care Hospital (2021030) and all individuals provided informed written consent.

**TABLE 1:** Hematological features, Hb electrophoresis of the couple.

Sample	Age/gender	Hb (g/L)	MCV (fL)	MCH (pg)	HbA	HbA2 (%)	HbF (%)
Wife	28/female	124.0	84.1	28.5	97.6	2.4	0
Husband	32/male	119.0	63.8	16.5	90.0	7.0	3.0

**FIGURE 1:**
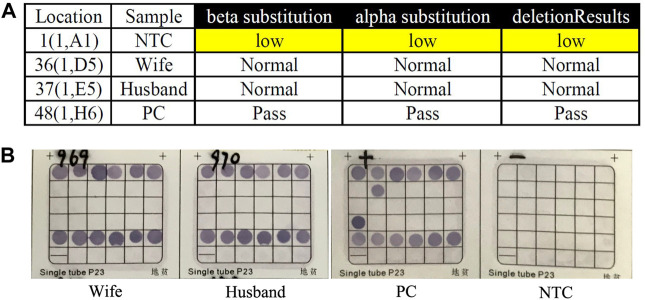
Routine genetic assays for 23 types of variations in HBA and HBB genes. **(A)** Auspension-array system was performed to detect 23 types of common variations in thalassemia. **(B)** Reverse dot blot (RDB) assay was used to detect 23 types of common variations in thalassemia (PC was β^17M/N^ and α^–SEA/N^). No routine variants in thalassemia had been detected in this couple by this two independent platforms (NTC: No Template Control; PC: Positive Control; Pass: Quality Control Passed).

**FIGURE 2:**
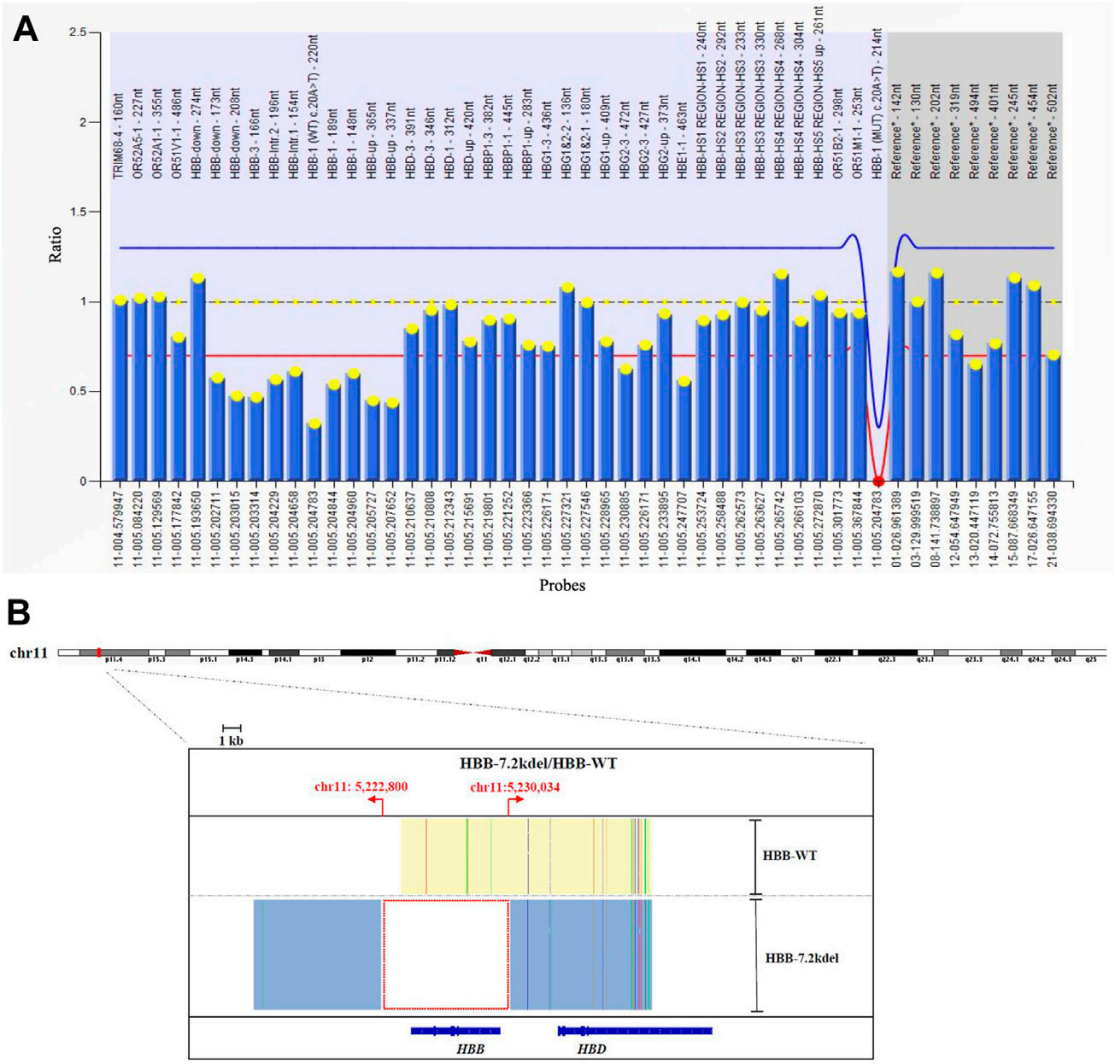
MLPA and TGS results. **(A)** MLPA result showed the deletion across 10 probes targeting HBB gene. (deletion probes involved 10 probes between hg38 loc. 11p15.4: 5224905-5229846, including HBB-up, HBB-1, HBB-1 (WT) c., HBB-Intr.1, HBB-Intr.2, HBB-3 and HBB-down regions). **(B)** TGS result showed the exact length of the deletion was 7.2 Kb (Chr11:5222800-5230034, hg38) located at HBB gene.

**FIGURE 3:**
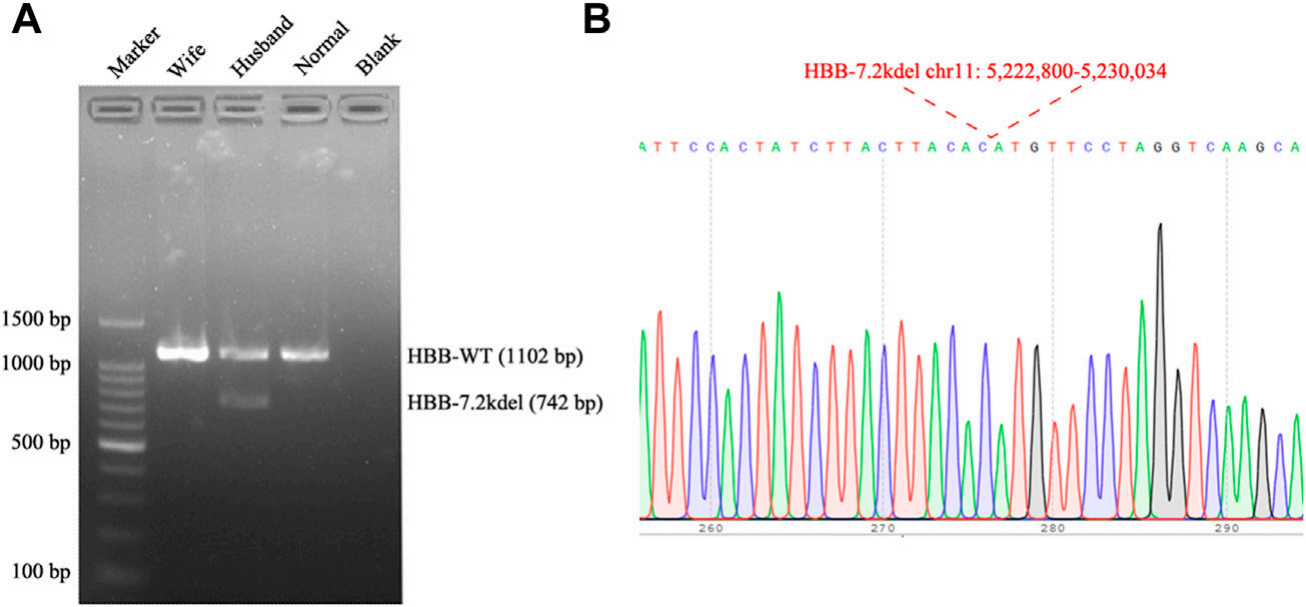
Gap PCR and Sanger sequencing results. **(A)** Gap PCR and agarose gel electrophoresis were performed to detect the deletion breakpoint. With the primers (7.2 Kb deletion forward primer: 5′-ATT​TCT​CCC​TTG​ATA​GTT​TCT​ACT​TTG​GGT​T-3′, Wild Type forward primer: 5′-TAC​CAT​CAT​CCT​GGC​TTC​AAG​GC-3′ and the common reverse primer: 5′-ATC​TGA​AGA​CTG​TAC​CTC​TGC​TCT​CC-3′), an unique fragment about 742 bp in length was only amplified in the husband (Normal: Normal individual; Blank: Water). **(B)** The fragment was then sequenced, the breakpoint was accordant with the TGS result.

Genomic DNA was extracted from the whole blood sample using automatic nucleic acid extraction instrument (Lab-Aid 824, Zhishan Biotechnology Co., Ltd., Xiamen, China). Thalassemia genotyping analysis were used two independent platforms: suspension-array system (Yin et al., 2014) and a reverse dot blot (RDB) assay thalassemia gene chip combined with flow-through hybridization technology platform (Chaozhou Hybribio Limited Corporation, China) (Lin et al., 2012). All this two assays were performed according to the manufacturer’s protocol. 23 genotypes of most common thalassemia had been detected, including 3 types of α-globin (HBA) genes deletions (–SEA, -α3.7, -α4.2), 3 types of point mutations in α-globin genes (α^CS^α, α^QS^α and α^WS^α) and 17 types of point mutations β-globin (HBB) genes (41–42M/N, 654M/N, -28M/N, 71–72M/N, 17M/N, β^E^M/N, 43M/N, −29M/N, 31M/N, −32M/N, IVS-I-1M, 27/28M, -30M, 14–15M, CAPM, IntM and IVS-I-5M). However, no variants had been detected of the above 23 genotypes of thalassemia in this couple (Figure 1). Considering the obvious abnormality of hematological phenotype in the husband, multiplex ligation-dependent probe amplification (MLPA, MRCHolland, Amsterdam, Netherlands) was utilized to test deletions and duplications within the β-globin genes. MLPA detected a deletion in the β-globin gene cluster (deletion probes involved 10 probes between hg38 loc. 11p15.4: 5224905-5229846, including HBB-up, HBB-1, HBB-1 (WT) c., HBB-Intr.1, HBB-Intr.2, HBB-3, and HBB-down regions, Figure 2A). However, the exact breakpoint location was not determined. Therefore TGS was adopted to characterize the deletion. Then, consistent with the MLPA result, TGS analysis confirmed a novel 7.2 Kb deletion (Chr11:5222800-5230034, hg38) at β-globin gene, and this deletion removed all of the HBB gene (Chr11:5225464-5227197, hg38) (Figure 2B). Ultimately, this novel deletion was further verificated by Gap PCR and Sanger sequencing (Figure 3).

## Discussion and conclusion

Although variations in the β-thalassemia was mainly due to point mutations, several large deletions had been described in recent years (Zhu et al., 2019). Due to the technical limitations, many breakpoints of large deletions in the β-globin gene cluster were not confirmed (Rooks et al., 2012). Research had shown that there were multiple cis-elements located in the β-globin gene cluster that could regulate the expression of the globin genes (Thein, 2013; Zhu et al., 2019). Therefore, ascertaining the breakpoints of deletions was important for hemoglobinopathy research (Hu et al., 2016). Furthermore, it was difficult to studies of molecular structure of these large deletions especially the accurate breakpoints, until the invention of TGS technology which could relatively accurately estimate the deletion range. TGS conducting on the PacBio sequencing platform, had shown significant advantages such as extra-long reads (80 Kb) and no requirement for PCR, had been used for thalassemia carrier screening in the last 2 years (Xiao and Zhou, 2020; Liang et al., 2021; Luo et al., 2022).

In this study, different methods were used for thalassemia carriers screening. First, routine hematological phenotypes of thalassemia were detected (MCV, MCH, and Hb%). Subsequently, genetic genotypes for 23 genotypes of common thalassemia variations, MLPA, TGS, and Gap PCR analyses for known and unknown genotypes were carried out. MLPA found a deletion in the β-globin gene cluster but it could not make clear the exact location of the breakpoint. Finally, a novel 7.2 Kb heterozygous deletion in β-globin gene cluster (Chr11:5222800-5230034, hg38) was confirmed by TGS and Gap PCR, which was consistent with the MLPA study. This novel 7.2 Kb heterozygous deletion in β-globin gene included all the functional HBB gene, corresponding to high Hb A2 level (7.0%), high Hb F level (3.0%) and low MCV(63.8 fl).

In conclusion, our studies showed that TGS technology was a powerful tool for thalassemia breakpoint detection, had promising potentiality in genetic screening of novel HBA1/2 and HBB variants that might be outside the scope or difficult to be accurately detected by traditional analysis methods, especially for the novel deletions in globin genes. Furthermore, MLPA combining with TGS analysis should be considered to perform an accurate diagnosis in uncertain thalassemia cases, which could also improve the accuracy of genetic counseling.

## Data Availability

The datasets for this article are not publicly available due to concerns regarding participant/patient anonymity. Requests to access the datasets should be directed to the corresponding author.
